# Metformin improves mitophagy-related pathways and mitochondrial-lysosomal homeostasis in chromosome 21 trisomic fibroblasts

**DOI:** 10.1007/s00018-026-06355-2

**Published:** 2026-07-22

**Authors:** Nunzia Mollo, Rosalba Natale, Morena D’Ariano, Simona Coppola, Daniele Di Meglio, Adriana Limone, Gaetano Calì, Claudia D’Agostino, Nunzia Pastore, Daniela Sarnataro, Simona Paladino, Anna Conti, Lucio Nitsch, Antonella Izzo

**Affiliations:** 1 Dept. of Molecular Medicine and Medical Biotechnology, Federico II University, Naples, Italy; 2 Istituto degli Endotipi in Oncologia, Metabolismo e Immunologia “G. Salvatore” (IEOMI), National Research Council (CNR), Naples, Italy; 3 Telethon Institute of Genetics and Medicine (TIGEM), Pozzuoli, NA Italy; 4 Dept. of Translational Medicine, Medical Genetics, Federico II University, Naples, Italy

**Keywords:** Mitophagy, Trisomy 21, Metformin, PINK1-PARKIN pathway, Mitochondrial quality control

## Abstract

Mitochondrial dysfunction is a core cellular abnormality in Down syndrome (DS), yet the mechanisms underlying impaired mitochondrial quality control remain poorly defined. Here, we provide an integrated characterization of the autophagy–mitophagy–lysosome axis in human trisomic fetal fibroblasts (DS-HFFs). We show that trisomic cells display a paradoxical combination of elevated basal autophagy/mitophagy-related markers and defective mitochondrial clearance, indicative of a saturated and inefficient quality control system. DS-HFFs exhibit increased steady-state levels of PINK1, PARKIN, OPTN, NDP52, LC3-II, and p62, accumulation of autophagic vacuoles, reduced autophagic flux, and impaired delivery of mitochondria to lysosomes. We further demonstrate that metformin alleviates the saturation of the autophagy–mitophagy pathway by modulating multiple components of the mitochondrial quality control pathway, including attenuation of mTOR-associated signaling, enhancement of autophagic flux and normalization of mitophagy-related protein levels. Crucially, metformin increases mitochondria–lysosome association and rescues lysosomal degradative competence as shown by increased Dq-BSA activity, and increased maturation of Cathepsin D. Together, our findings support mitophagy inefficiency due to pathway saturation as a key pathogenetic mechanism in trisomic cells and identify metformin as a pharmacological tool capable of relieving the proteostatic overload intrinsic to trisomy 21. More broadly, these results align with a model in which aneuploidy imposes a chronic proteostatic burden that compromises organelle quality control.

## Introduction

Down syndrome (DS) is characterized by intellectual disability, distinctive physical features, and an increased risk of neurodegenerative, cardiovascular, and metabolic disorders [1]. Mitochondrial dysfunction plays a central role in cellular homeostasis, influencing tissue and organ physiology, and is increasingly recognized as a major contributor to the pathogenesis of various non-communicable diseases [2], many of which are highly prevalent in individuals with DS [3].

A growing body of evidence from DS cells, tissues, and animal models supports a critical involvement of mitochondrial dysfunction in DS pathophysiology [4, 5]. Trisomy 21 (T21) cells display structural and functional mitochondrial abnormalities, including altered mitochondrial morphology, impaired bioenergetics, and accumulation of damaged mitochondria [4, 6]. Rather than representing a single primary defect, mitochondrial dysfunction in DS is increasingly viewed as part of a self-reinforcing cycle in which altered mitochondrial dynamics and impaired turnover emerge from a chronic proteostatic stress that progressively saturates cellular quality control systems.

The accumulation of damaged mitochondria may arise, at least in part, from a functional bottleneck in mitochondrial clearance through mitophagy, a selective, multistep autophagic process responsible for the removal of dysfunctional mitochondria [7]. Mitophagy is essential for maintaining mitochondrial quality and, together with mitochondrial biogenesis and dynamics, contributes fundamentally to cellular homeostasis [8]. Defective mitophagy has been increasingly implicated in a range of human diseases, particularly neurodegenerative disorders, where impaired clearance of damaged mitochondria contributes to cellular vulnerability and disease progression [9–12].

The PINK1–PARKIN pathway is the best-characterized regulator of mitophagy [13–16]. Upon mitochondrial depolarization, PTEN-induced putative kinase 1 (PINK1) accumulates on the outer mitochondrial membrane of damaged organelles and recruits the E3 ubiquitin ligase Parkin. Parkin-mediated ubiquitination promotes the engagement of autophagic cargo receptors, including p62/sequestosome 1 (SQSTM1), optineurin (OPTN), and nuclear dot protein 52 kDa (NDP52), which facilitate the sequestration of damaged mitochondria into autophagosomes [17]. Additional autophagic components, including members of the ATG8 family, such as microtubule-associated proteins 1 light chain 3 (LC3), participate in autophagosome formation and maturation.

In parallel, the mechanistic target of rapamycin (mTOR) acts as a central inhibitor of autophagy, integrating nutrient and energetic cues to regulate autophagosome biogenesis [18]. Notably, abnormally elevated mTOR signaling has been consistently reported in DS cellular and animal models, and hyperactivation of this pathway has emerged as a key molecular alteration in trisomic cells, highlighting mTOR as a relevant contributor to impaired autophagy and mitochondrial quality control axis in DS [19–24]. Following sequestration, damaged mitochondria are delivered to lysosomes for degradation, a step dependent on lysosomal membrane proteins such as LAMP1 and proteolytic enzymes like cathepsin D, which ensure efficient clearance of the mitophagic cargo and maintenance of mitochondrial quality [25].

Previous work from our group demonstrated substantial mitochondrial impairment in human DS fetal fibroblasts (DS-HFFs), characterized by a highly fragmented mitochondrial network and increased mitochondrial number, associated with altered expression of fission/fusion-machinery proteins [26–29]. These defects were linked to overexpression of chromosome 21 genes and miRNAs that disrupt mitochondrial homeostasis, bioenergetics, and biogenesis [4, 30, 31]. We showed that the chromosome 21 gene *NRIP1* negatively regulates PGC-1α, the master activator of mitochondrial biogenesis [27], and that targeting the NRIP1–PGC-1α axis with metformin, a widely used anti-diabetic drug, restored mitochondrial network integrity and improved mitochondrial function in DS-HFFs [29]. However, metformin is also known to activate AMPK, reduce oxidative stress, and inhibit mTOR signaling [9, 32], suggesting that it may furtherly influence mitochondrial quality control by modulating autophagy-related pathways.

Based on this evidence, we hypothesized that impaired mitophagy contributes to the accumulation of dysfunctional mitochondria in trisomic fibroblasts and that metformin may counteract the mitochondrial dysfunction by restoring the autophagy-mitophagy pathway as well as by regulating the mitochondrial biogenesis.

In this study, we investigated the status of mitophagy in DS-HFFs and examined the effects of metformin on key elements of mitochondrial clearance, including the PINK1–PARKIN axis, autophagic cargo receptors, autophagic flux, and lysosomal degradation. By integrating biochemical and imaging-based approaches, we aimed to define whether defects in mitophagy efficiency contribute to mitochondrial dysfunction in trisomic cells and to assess the extent to which metformin can modulate these processes.

## Materials and methods

### Ethics statement

Human fetal fibroblasts were obtained from the “Telethon Bank of Fetal Biological Samples” at the University of Naples Federico II. All protocols related to the Bank were approved by the “Ethics Committee University Federico II”.

Five samples were derived from skin biopsies of human fetuses with trisomy of chromosome 21 (DS-HFFs), and three samples were obtained from skin biopsies of euploid fetuses (N-HFFs) following therapeutic abortion at 18–22 weeks of gestation.

### Samples

Fibroblasts were cultured in T25 flasks (Corning Incorporated-Life Sciences, New York, USA) in Dulbecco’s Modified Eagle Medium (DMEM) supplemented with 10% fetal bovine serum (FBS; Merck, Darmstadt, Germany) and 1% penicillin/streptomycin (Gibco™, Thermo Fisher Scientific, Waltham, MA, USA). Cells were maintained at 37 °C in a humidified atmosphere containing 5% CO₂. Karyotype analysis was periodically performed following standard procedures [33].

### Mitophagy study

To induce mitochondrial depletion via membrane depolarization, cells were treated with 20 µM Carbonyl cyanide m-chlorophenyl hydrazone (CCCP; Sigma, C2759) for 6 h and/or 0.1 µM bafilomycin A1 (BAF; Sigma, B1793), a specific inhibitor of the lysosomal vacuolar-type H⁺-ATPase (V-ATPase) [25], for 6 h. Control cells received an equivalent volume of DMSO.

### Metformin treatment

Metformin hydrochloride (Cayman Chemical, Cat. No. 13118) was dissolved in water to prepare a 100 mM stock solution. Cells were treated by adding metformin to the culture medium at final concentrations of 0.5 mM, 2.5 mM, or 5 mM. Fresh metformin-containing medium was refreshed every 24 h for a total of 72 h. Control cells received an equivalent volume of solvent only.

### Western blotting

Western blotting was performed as previously described [29]. Protein extracts, separated by sodium dodecyl sulphate - polyacrylamide gel electrophoresis (SDS-PAGE) and transferred onto PVDF Transfer Membranes, were incubated with the following specific primary antibodies: anti-LC3B (1:1000, #2775, Cell Signaling); anti-NDP52 (1:1000, 12229-1-AP, Proteintech); anti-phospho-OPTINEURIN (Ser177) (1:1000, #57548, Cell Signaling); anti-OPTINEURIN (C-2) (1:1000, sc-166576, Santa Cruz Biotechnology); anti-PINK1 (1:1000, 23274-1-AP, Proteintech); anti-PARKIN (PRK8) (1:500, #4211, Cell Signaling); anti-SQSTM1/p62 (C-terminus) (1:1000, GP62-C, Progen); anti-phospho-mTOR (Ser2448) (1:1000, #2971, Cell Signaling); anti-mTOR (1:1000, #2983, Cell Signaling); anti-LAMP1 (D4O1S) (1:1000, #15665, Cell Signaling); anti-CATHEPSIN D (1:1000, PA5-72182, Invitrogen); anti-Phospho-AMPKα (Thr172)(40H9) (1:1000, #2535, Cell Signaling); AMPKα (1:1000; #2532; Cell Signaling); Phospho-ATG14 (Ser29)(D4BM8) (1:1000, #92340, Cell Signaling); ATG14 (D1A1N) (1:1000, #96752, Cell Signaling); Phospho-ULK1 (Ser555)(D1H4) (1:1000, #5869, Cell Signaling); ULK1 (D8H5) (1:1000, #8054, Cell Signaling); anti-α-TUBULIN (DM1A) (1:10000, ab7291, Abcam); anti-CALNEXIN (1:1000, ADI-SPA-860, Enzo); anti-GAPDH (6C5) (1:1000, sc-32233, Santa Cruz Biotechnology). Primary antibodies were detected with the appropriate HRP-conjugated secondary antibodies (Santa Cruz Biotechnology or GE-Healthcare) and revealed by chemiluminescence (Pierce) using digital imaging on a Bio-Rad ChemiDoc XRS apparatus or Fuji X-ray film.

### Immunofluorescence and confocal microscopy

For LC3 and TOM20/LC3 staining, cells were fixed in methanol at −20 °C for 5 min and permeabilized for 5 min in 0,2% Triton X-100 in PBS. Blocking was performed with 1% bovine serum albumin (BSA) in PBS for 1 h at room temperature. Cells were incubated with primary antibodies anti-LC3B (E5Q2K) (1:200, #83506, Cell Signaling) and anti-TOM20 (D8T4N) (1:100; #42406, Cell Signaling) o/n at 4 °C, washed in 0.5% BSA in PBS, and then incubated with secondary antibodies for 30 min.

For TOM20/p62 staining, cells were fixed as previously described [34] and permeabilized for 5 min in 0,2% Triton X-100 in PBS. Blocking was performed with 1% bovine serum albumin (BSA) in PBS for 1 h at room temperature. Cells were incubated with primary antibodies anti-SQSTM1/p62 (D5L7G) (1:100, #88588, Cell Signaling) and anti-TOM20 (D8T4N) (1:100; #42406, Cell Signaling) o/n at 4 °C, washed in 0.5% BSA in PBS, and then incubated with secondary antibodies for 30 min.

For TOM20/LysoTracker Red staining, cells cultured on 12-mm diameter glass coverslips were incubated with 0.1 µM LysoTracker™ Red DND-99 (Thermo Fisher, L7528) for 1 h in complete culture medium before fixing. Cells were permeabilized for 5 min in 0,2% Triton X-100 in PBS and blocked with 1% bovine serum albumin (BSA) in PBS for 1 h at room temperature. Cells were incubated with the primary antibody anti-TOM20 (D8T4N) (1:100; #42406, Cell Signaling) for 2 h in humidified chamber, washed in 0.5% BSA in PBS, and then incubated with secondary antibody for 30 min.

For TFEB staining, cells were fixed in paraformaldehyde 4% (PFA 4%) at room temperature for 20 min. Blocking and permeabilization were performed with 3% bovine serum albumin (BSA) and 5% goat serum in 0,1% Triton X-100 in PBS for 1 h at room temperature. Cells were incubated with primary antibodies anti-TFEB (1:100, #4240, Cell Signaling) o/n at 4 °C, washed in 0.5% BSA in PBS, and then incubated with secondary antibodies for 30 min.

The secondary antibodies used were Alexa Fluor^®^ 488 goat anti-mouse IgG (H + L) (Thermo Fisher Scientific) (green) and DyLight^®^ 550 goat anti-rabbit IgG (H + L) (ImmunoReagents, Raleigh, NC, USA) (red). Nuclei were stained with the DNA intercalator DAPI (1:1000 in PBS) for 10 min. Coverslips were finally mounted in 50% glycerol in PBS.

All immunofluorescence experiments were performed at least in duplicate. Images were collected using the LSM 700 confocal laser scanning microscope (Carl Zeiss, Jena, Germany) equipped with a Plan Apo×63 oil immersion objective lens (NA 1.4); diode lasers (at 405, 488, and 555 nm) were used as light sources. Images were acquired with the confocal pinhole set to one Airy unit using the same setting (laser power, detector gain, and threshold of fluorescence intensity) in all experimental conditions. Three-dimensional reconstructions of Z-slices collected from the top to the bottom of the cells were carried out using Zeiss ZEN Black software. Thirty or fifty random single cells were analyzed for each imaging analysis using Fiji.

### Image analysis

Quantification and colocalization analyses were performed using ImageJ/Fiji software. Z-stack images were first projected using maximum intensity projection to retain relevant fluorescence signals across the optical sections. Regions of interest (ROIs) encompassing entire cells were manually drawn, and mean fluorescence intensities as well as the number of fluorescent puncta were measured and corrected for background signal.

Colocalization analysis was performed to assess the spatial overlap between TOM20 and LC3, TOM20 and p62, and TOM20 and LysoTracker. The degree of colocalization was quantified using the thresholded Manders´ coefficients, calculated within the defined ROIs by the JaCoP plugin in ImageJ [35]. Manders’ coefficients represent the fraction of the total intensity of one channel that spatially overlaps with the other. Coefficients were calculated on thresholded images to exclude background signal and range from 0 to 1, independently of linear intensity correlation.

To define co-localization regions, images were segmented using the Otsu thresholding algorithm, allowing identification of pixels corresponding to overlapping signals of the reference proteins. This approach ensures unbiased quantification of co-localized structures within the three-dimensional datasets acquired from Z-stack images.

### Dq-BSA and magic red assays

Lysosomal degradative activity was assessed using Dq-Red-BSA (Thermo Fisher Scientific), a self-quenched fluorescent substrate that emits fluorescence upon proteolytic degradation within lysosomes and autolysosomes, and the Magic Red Cathepsin B assay (ImmunoChemistry Technologies), a fluorogenic substrate that reports cathepsin B enzymatic activity in live cells. Cells were cultured for 4 days and treated with metformin at a concentration of 2.5mM for the last 72 h. Following drug treatment, cells were incubated overnight with 10 µg/mL Dq-Red-BSA in complete culture medium. For the Magic Red assay, cells were incubated with the Magic Red reagent according to the manufacturer’s instructions immediately prior to imaging.

Live-cell image acquisition was performed using an Opera Phenix High-Content Screening System (Revvity) equipped with a 40× water-immersion objective. Cells were seeded in 96-well PhenoPlates (Revvity, Cat. No. 6055302). Multiple fields were acquired per well, and the number of analyzed fields and cells for each experiment was recorded within the corresponding Signal Image Artist (SiMa, Revvity) analysis project.

Image analysis was performed using Signals Image Artist (SiMa, Revvity). For the Dq-BSA assay, fluorescent puncta were identified using an intensity-based thresholding algorithm, and the number of puncta was normalized to the total number of nuclei detected in each well to obtain the Dq-BSA spots-per-cell parameter. Compounds were considered active when the mean Dq-BSA spots-per-cell value exceeded the mean of control cells by more than three standard deviations and reached statistical significance (*p* ≤ 0.01). For the Magic Red assay, lysosomal cathepsin B activity was quantified by measuring the fluorescence intensity and/or the number of Magic Red-positive vesicular structures on a per-cell basis after nuclear normalization.

### Statistical procedures

Unless otherwise stated, all experiments were performed independently and in duplicate. Statistical analyses were conducted using GraphPad Prism version 5.0 (GraphPad Software, La Jolla, CA, USA; http://www.graphpad.com; last accessed 2010). Student’s t-test was used to evaluate the significance of differences between groups. A p-value of < 0.05 was considered statistically significant.

## Results

### Trisomic fibroblasts exhibit dysregulated mitophagy-related pathways and impaired autophagic flux and clearance

**Fig. 1:**
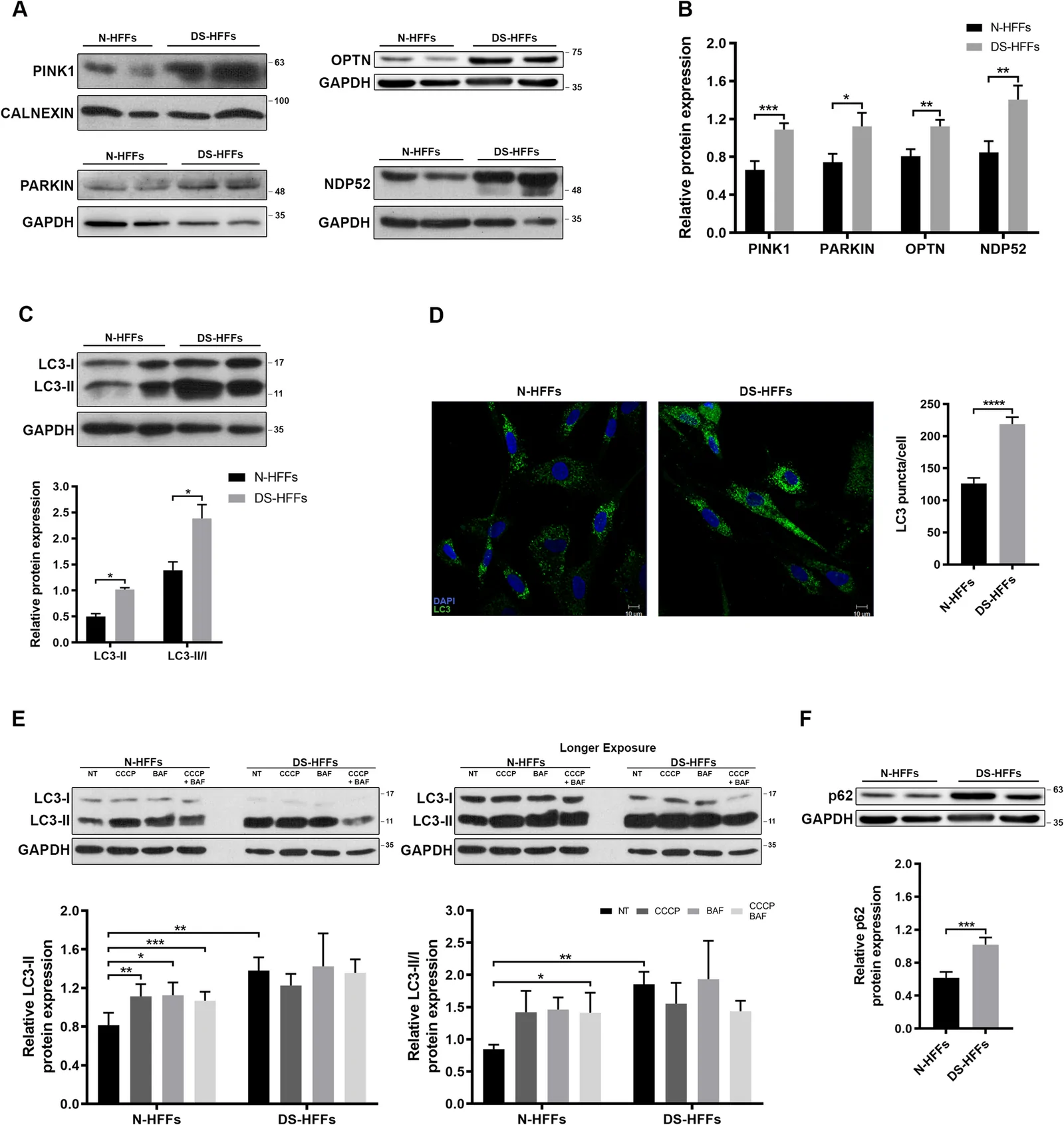
Mitophagy-related pathways and autophagy flux are impaired in trisomic cells.** (A)** Representative immunoblots of PINK1, PARKIN, OPTINEURIN and NDP52 expression in N-HFFs and DS-HFFs. **(B)** Densitometric quantification from at least three independent experiments. **(C)** Representative immunoblot showing LC3 (LC3-I and LC3-II) in N-HFFs and DS-HFFs. The graphs illustrate densitometric quantification of LC3-II/GAPDH and LC3-II/I ratio from at least three independent experiments. **(D)** Representative immunofluorescence images of LC3 staining (green) in N-HFFs and DS-HFFs. Nuclei were counterstained with DAPI (blue). The graph illustrates quantification of LC3-positive puncta per cell. Analyses were performed on two independent euploid and two trisomic cultures, each assessed in duplicate. Thirty randomly selected cells per sample were evaluated. **(E)** Representative immunoblots showing LC3 (LC3-I and LC3-II) in basal conditions (NT) and after treatment with CCCP (20 µM), bafilomycin A1 (BAF, 0,1 µM), or CCCP + BAF. A longer exposure of the same blot is shown to highlight the LC3-I band. The graphs illustrate densitometric quantification of LC3-II/GAPDH and LC3-II/I ratio from four different experiments. **(F)** Representative immunoblot showing p62 expression in N-HFFs and DS-HFFs. The graph illustrates densitometric quantification from at least three independent experiments. CALNEXIN and GAPDH were used as loading controls. Data are expressed as mean ± SEM of DS-HFFs relative to N-HFFs. N-HFFs: euploid fetal fibroblasts; DS-HFFs: trisomic fetal fibroblasts. *p ≤ 0.05; **p ≤ 0.01; ***p ≤ 0.001; ****p ≤ 0.0001

Mitophagy impairment contributes to the pathogenesis of several neurodegenerative and metabolic disorders, reflecting its central role in mitochondrial quality control [10]. To evaluate whether T21 alters the mitophagic machinery, we quantified key mediators of the PINK1-PARKIN mitophagy pathway. Compared with euploid controls, DS-HFFs displayed significantly increased steady-state levels of PINK1 and PARKIN (Fig. 1A, B), indicative of pathway dysregulation and consistent with impaired turnover of mitophagy-related components.

The cargo receptors OPTN and NDP52, which mediate recruitment of autophagic machinery to ubiquitinated mitochondria [17], were both significantly increased in DS-HFFs (Fig. 1A, B), consistent with accumulation of downstream mitophagy-related components. However, as shown below, these changes do not translate into efficient mitochondrial clearance, suggesting saturation or inefficiency at later stages of the pathway.

We next examined markers of autophagosome formation and turnover. Under basal conditions, DS-HFFs displayed higher LC3-II levels than euploid controls (Fig. 1C), calculated both as LC3-II/GAPDH and LC3-II/I ratios, consistent with the increased number of LC3-positive puncta observed by confocal imaging (Fig. 1D), indicating expansion of the autophagosomal compartment.

Accumulation of LC3-II and LC3 puncta can arise from two non-exclusive mechanisms: enhanced autophagosome formation or impaired degradation [36]. To distinguish between these possibilities, we directly assessed autophagic flux. We treated cells with the mitochondrial uncoupler carbonyl cyanide m-chlorophenyl hydrazone (CCCP, 20 µM) and the lysosomal inhibitor bafilomycin A1 (BAF, 0.1 µM) for 6 h [37]. First, the increased LC3-II levels observed in trisomic cells were confirmed also in the presence of BAF (Fig. 1E). Treatment with CCCP triggered a robust increase in LC3-II in euploid fibroblasts, consistent with activation of autophagosome formation in response to mitochondrial stress (Fig. 1E). In contrast, DS-HFFs showed minimal LC3-II accumulation following CCCP exposure, indicating a blunted autophagic response.

Similarly, BAF treatment alone, or in combination with CCCP, resulted in a marked LC3-II accumulation in N-HFFs but a significantly reduced response in DS-HFFs. Quantitative evaluation of LC3-II differences in the presence and absence of BAF in each cell type (Fig. 1E) confirmed a reduction in autophagic flux in DS-HFFs, supporting the conclusion that autophagosomes are inefficiently cleared.

Finally, we assessed the levels of p62, a selective autophagy substrate degraded upon autophagosome–lysosome fusion. Because p62 turnover is tightly coupled to autophagic degradation, its accumulation is widely used as a marker of defective autophagic flux [25, 38]. DS-HFFs exhibited markedly elevated p62 levels (Fig. 1F), indicating that autophagic degradation is insufficient to balance p62 synthesis, resulting in net accumulation.

Together, these data indicate that trisomic fibroblasts exhibit sustained engagement of mitophagy-related components but reduced autophagic turnover.

### Basal autophagosome–mitochondria association is increased in DS-HFFs but shows limited responsiveness to mitochondrial stress

**Fig. 2:**
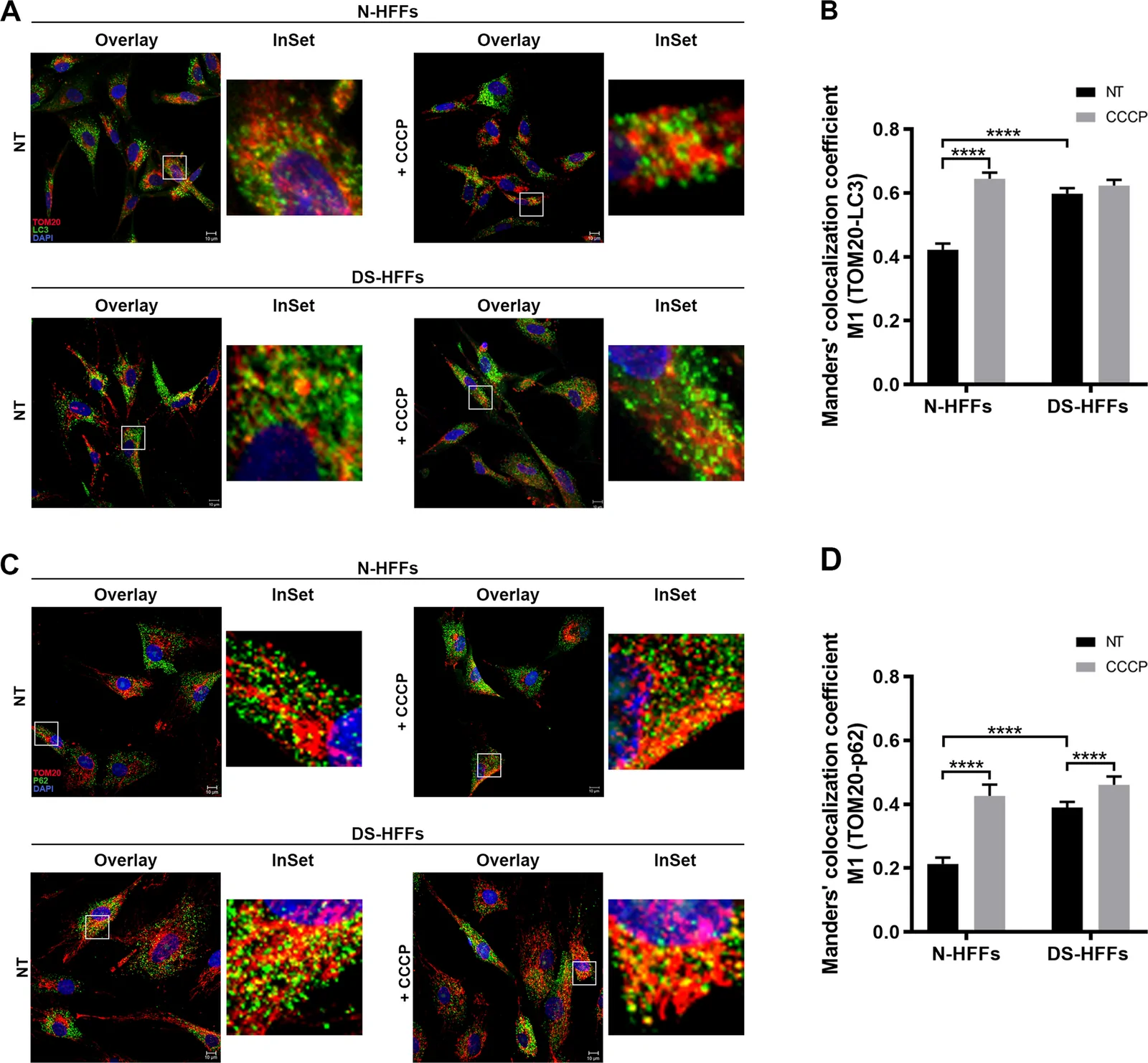
Colocalization of mitochondria with LC3- or p62-positive autophagosomes in euploid and trisomic fibroblasts.** (A)** Representative confocal images of N-HFFs and DS-HFFs stained for the mitochondrial marker TOM20 (red) and LC3 (green), and their relative Inset image, under basal conditions and after CCCP treatment (20 µM, 6 h). Nuclei are labelled with DAPI (blue). Scale bar: 10 μm **(B)** The graphs represent the Manders’ colocalization coefficient M1 of TOM20-LC3 overlap, calculated using the JACoP plugin of ImageJ. (**C**) Representative confocal images of N-HFFs and DS-HFFs stained for TOM20 (red) and p62 (green), and their relative Inset image, under basal conditions and after CCCP treatment. Nuclei are labelled with DAPI (blue). Scale bar: 10 μm **(D)** The graphs represent the Manders’ colocalization coefficient M1 of TOM20-p62 overlap, calculated using the JACoP plugin of ImageJ. The analysis was performed on three independent euploid and three trisomic cultures. Fifty randomly selected cells per sample were analyzed. N-HFFs: euploid fetal fibroblasts; DS-HFFs: trisomic fetal fibroblasts. ****p ≤ 0.0001

To assess potential alterations in mitophagy-related processes, we quantified the spatial association between mitochondria and autophagosomes by measuring TOM20-LC3 and TOM20-p62 colocalization. Under basal conditions, DS-HFFs displayed significantly higher colocalization than euploid fibroblasts (Fig. 2), consistent with an increased steady-state association of mitochondria within autophagosomal structures. This finding is in line with the elevated LC3-II and p62 levels observed above and suggests accumulation of mitophagy-related intermediates under resting conditions.

Upon CCCP treatment, euploid fibroblasts exhibited the expected increase in TOM20-LC3 and TOM20-p62 colocalization, consistent with enhanced recruitment of autophagic machinery to depolarized mitochondria. In contrast, DS-HFFs showed either a much lower (for p62) or insignificant (for LC3) further increase in colocalization beyond their already elevated basal levels **(**Fig. 2**)**, indicating limited dynamic responsiveness to mitochondrial stress.

These findings indicate that although damaged mitochondria are recognized and sequestered, further inducibility of the pathway is constrained in trisomic fibroblasts.

### DS-HFFs exhibit altered lysosomal association and reduced mitochondrial delivery to acidic compartments

**Fig. 3:**
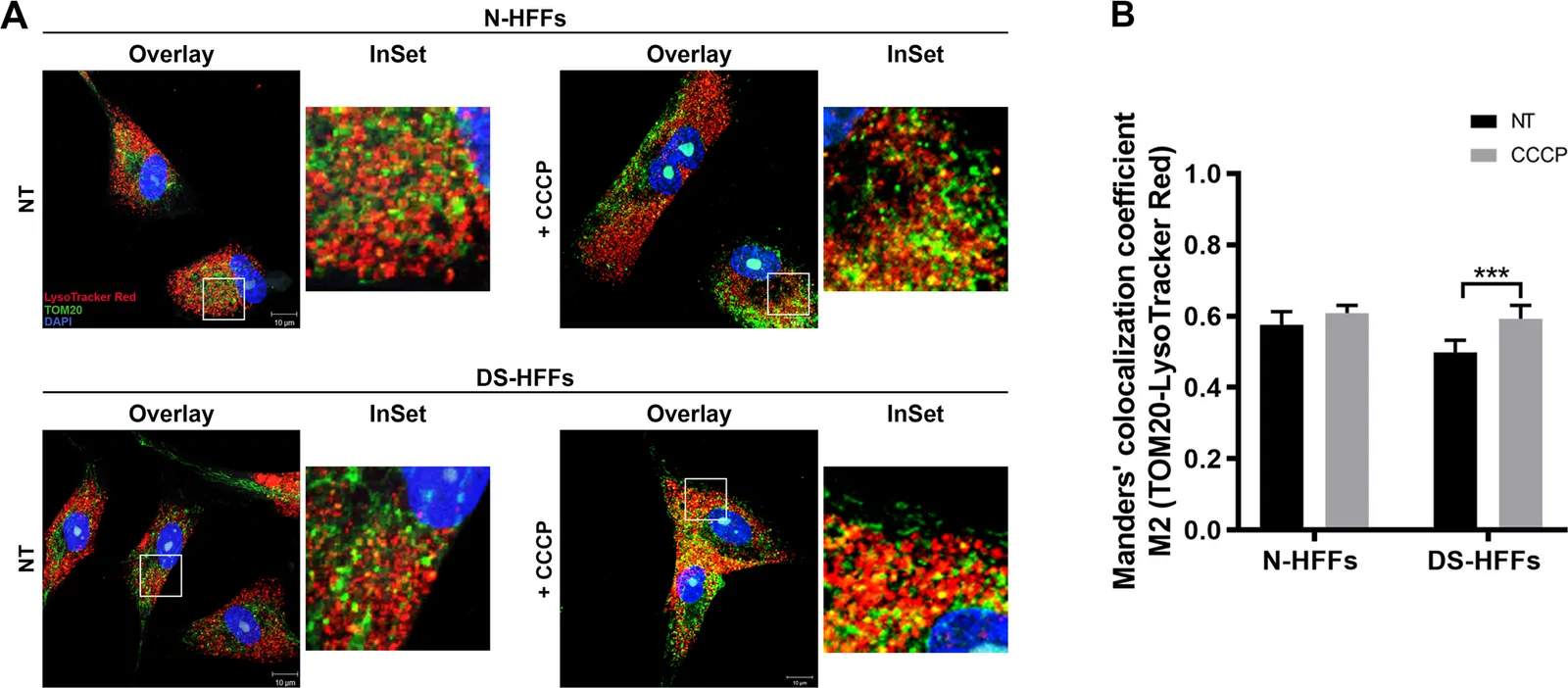
Colocalization of mitochondria with lysosomes in euploid and trisomic fibroblasts.** (A)** Representative confocal images of N-HFFs and DS-HFFs stained with the mitochondrial marker TOM20 (green) and LysoTracker Red, and the relative InSet image, under basal conditions and after CCCP treatment (20 µM, 6 h). Nuclei are labelled with DAPI (blue). Scale bar: 10 μm. **(B)** The graphs represent the Manders’ colocalization coefficient M2 of TOM20-LysoTracker Red overlap, calculated using the JACoP plugin of ImageJ. The analysis was performed on three independent euploid and three trisomic cultures. Fifty randomly selected cells per sample were analyzed. N-HFFs: euploid fetal fibroblasts; DS-HFFs: trisomic fetal fibroblasts. ***p ≤ 0.001

Because completion of mitophagy requires fusion with lysosomes, to assess the association between mitochondria and acidic compartments, we quantified TOM20–LysoTracker Red colocalization. Under basal conditions, DS-HFFs showed substantially reduced mitochondria–lysosome colocalization compared with euploid cells (Fig. 3), consistent with impaired late-stage delivery of mitochondria to acidic degradative compartments.

Upon CCCP-induced depolarization, trisomic fibroblasts exhibited increased TOM20–LysoTracker Red colocalization **(**Fig. 3**)**, indicating enhanced association between mitochondria and acidic compartments following mitochondrial stress.

These data localize the functional defect to late stages of the autophagy–lysosome axis.

### Metformin modulates mTOR-associated signaling and improves autophagic turnover in trisomic fibroblasts

**Fig. 4:**
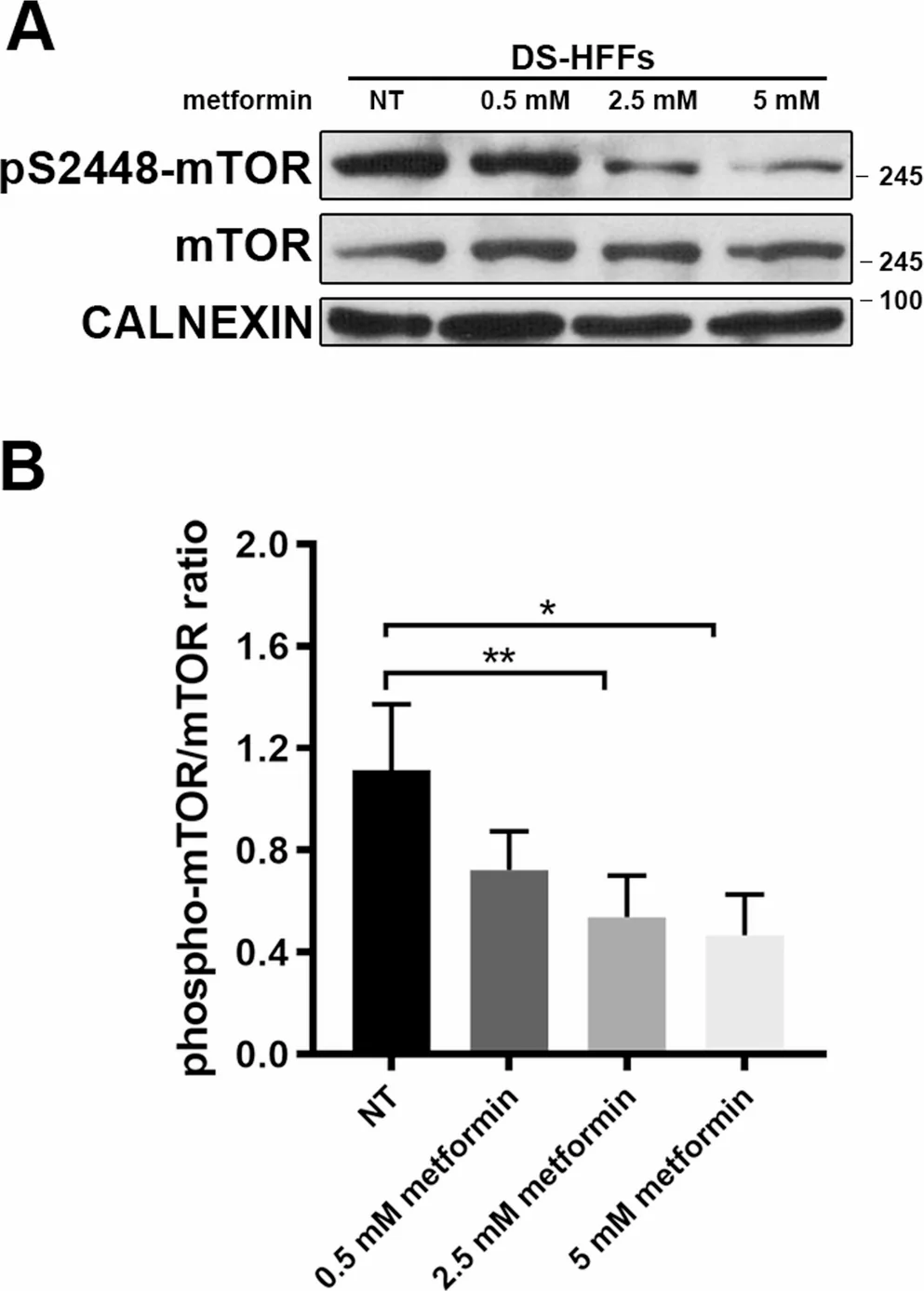
Metformin reduces mTOR phosphorylation at Ser2448 in trisomic fibroblasts.** (A)** Representative immunoblots showing total mTOR and phosphorylated mTOR (Ser2448) in DS-HFFs following metformin treatment. **(B)** Densitometric quantification from three independent experiments Data are expressed as mean ± SEM of three DS-HFFs cultures. CALNEXIN was used as a loading control. DS-HFFs: trisomic fetal fibroblasts. *p ≤ 0.05; **p ≤ 0.01

To assess whether metformin could counteract impaired autophagy in trisomic cells, DS-HFFs were treated with increasing concentrations of the drug (0.5, 2.5, and 5 mM) for 72 h and mTOR phosphorylation at Ser2448 - a key regulatory site within its catalytic domain - was evaluated. Metformin treatment elicited a clear dose-dependent reduction in Ser2448 phosphorylation relative to total mTOR levels (Fig. 4), consistent with modulation of mTOR-associated signaling in trisomic fibroblasts.

**Fig. 5:**
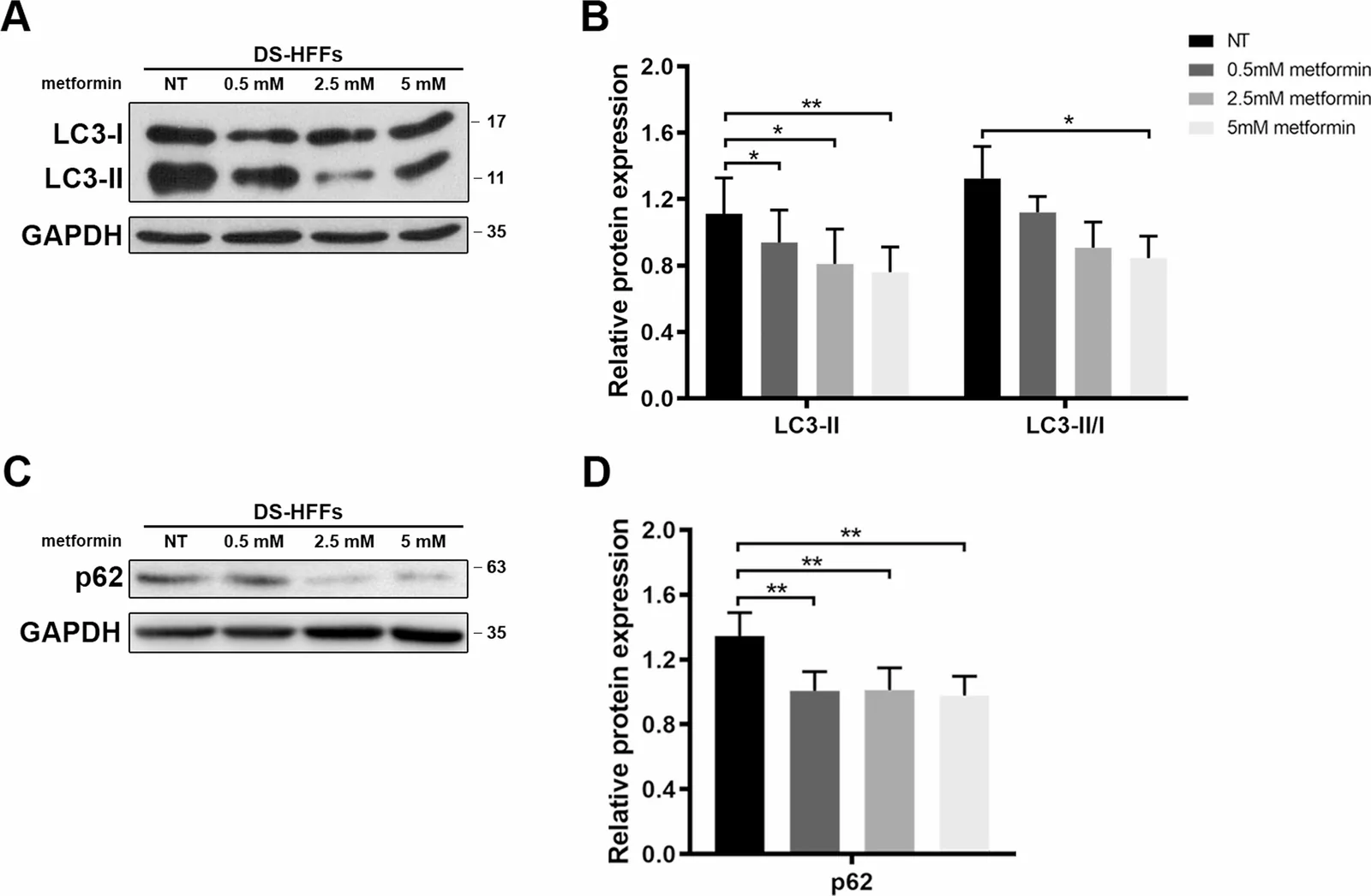
Metformin decreases LC3-II and p62 protein levels in trisomic fibroblasts.** (A)** Representative immunoblot showing LC3 (LC3-I and LC3-II) expression in DS-HFFs following metformin treatment. **(B)** Densitometric quantification of LC3-II/GAPDH and LC3-II/I ratio from at least five independent experiments. **(C)** Representative immunoblot showing p62 expression in DS-HFFs following metformin treatment. **(D)** Densitometric quantification of p62 expression from at least five independent experiments. Data are expressed as mean ± SEM of three DS-HFFs cultures. GAPDH was used as a loading control. DS-HFFs: trisomic fetal fibroblasts. *p ≤ 0.05; **p ≤ 0.01

While Ser2448 phosphorylation alone does not provide a complete measure of mTOR kinase activity, these changes occur in parallel with functional readouts of autophagic processing described below. Indeed, metformin treatment led to a marked decrease in LC3-II and p62 levels in DS-HFFs (Fig. 5), consistent with improved autophagosome clearance rather than reduced autophagy initiation.

### Metformin modulates mitophagy-related markers and enhances lysosomal competence in trisomic fibroblasts

**Fig. 6:**
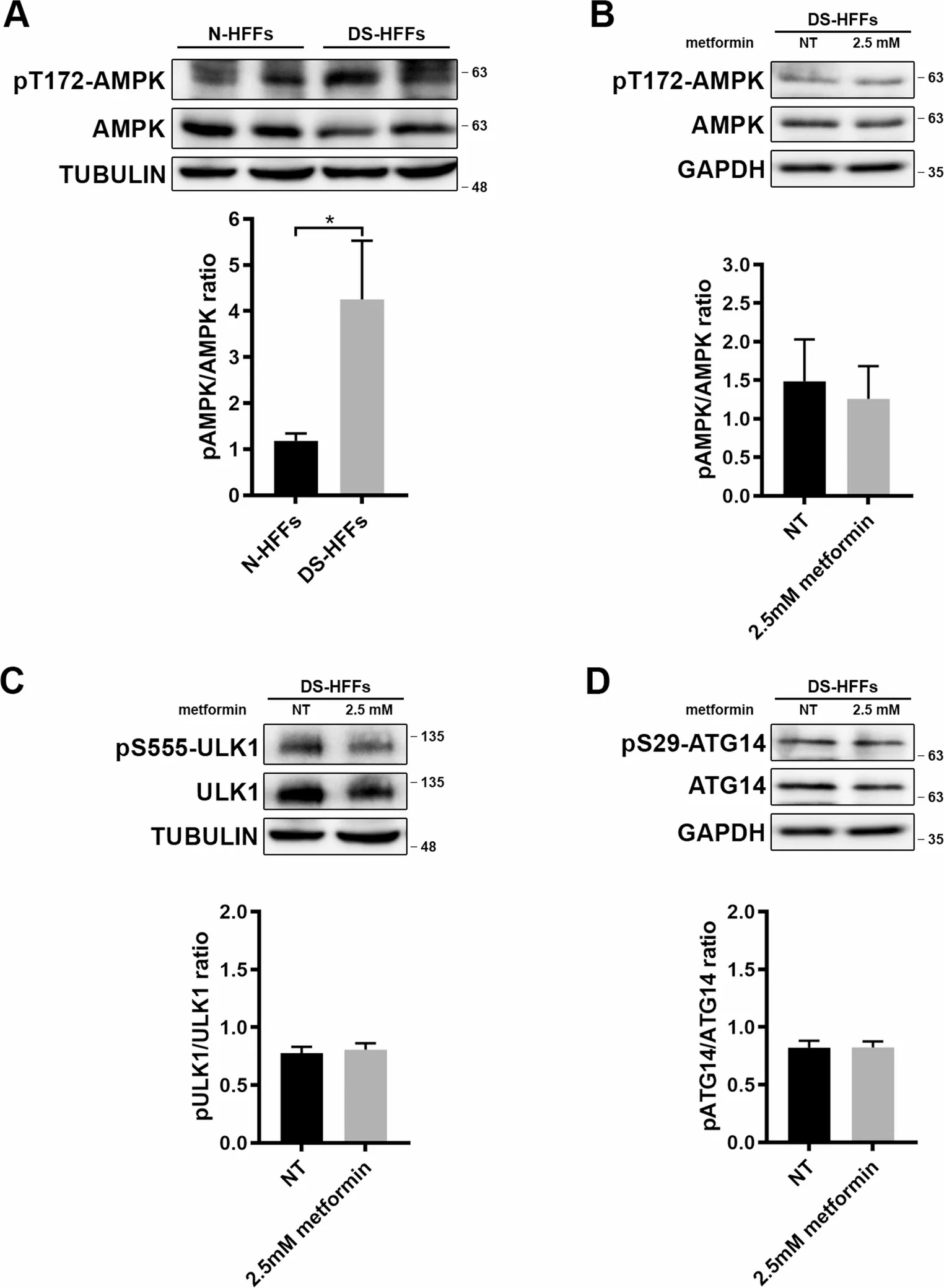
AMPK is hyperactivated in trisomic cells and not more activated after metformin.** (A)** Representative immunoblots showing total AMPK and phosphorylated AMPK (Thr172) expression in N-HFFs and DS-HFFs. The graph illustrates densitometric quantification from at least two independent experiments. **(B)** Representative immunoblots showing total AMPK and phosphorylated AMPK (T172) in DS-HFFs following metformin treatment. The graph illustrates densitometric quantification of pAMPK/AMPK ratio from at least two independent experiments. **(C)** Representative immunoblot showing total ULK1 and phosphorylated ULK1 (Ser555) in DS-HFFs following metformin treatment. The graph illustrates densitometric quantification of pULK1/ULK1 ratio from at least two independent experiments. **(D)** Representative immunoblot showing total ATG14 and phosphorylated ATG14 (Ser29) in DS-HFFs following metformin treatment. The graph illustrates densitometric quantification of pATG14/ATG14 ratio from at least two independent experiments. Data are expressed as mean ± SEM of two N-HFFs and two DS-HFFs cultures. TUBULIN and GAPDH was used as a loading control. N-HFFs: euploid fetal fibroblasts; DS-HFFs: trisomic fetal fibroblasts; *p ≤ 0.05

Given the evidence that metformin enhances autophagic turnover in trisomic fibroblasts, we next investigated whether this effect extends to the mitophagy-related pathway. Previous studies have reported that metformin promotes mitophagy in specific cellular contexts [39], supporting its role in mitochondrial quality control. Activation of the 5’ AMP-activated protein kinase (AMPK) is one of the major pathways regulated by metformin. We therefore measured AMPK activation in euploid and trisomic cells. We found that AMPK was already activated in DS-HFFs (Fig. 6A) and was not further activated after metformin treatment (Fig. 6B). We also evaluated the main AMPK substrates, ULK1 and ATG14, but no changes were detected after drug treatment (Fig. 6C, D).

**Fig. 7:**
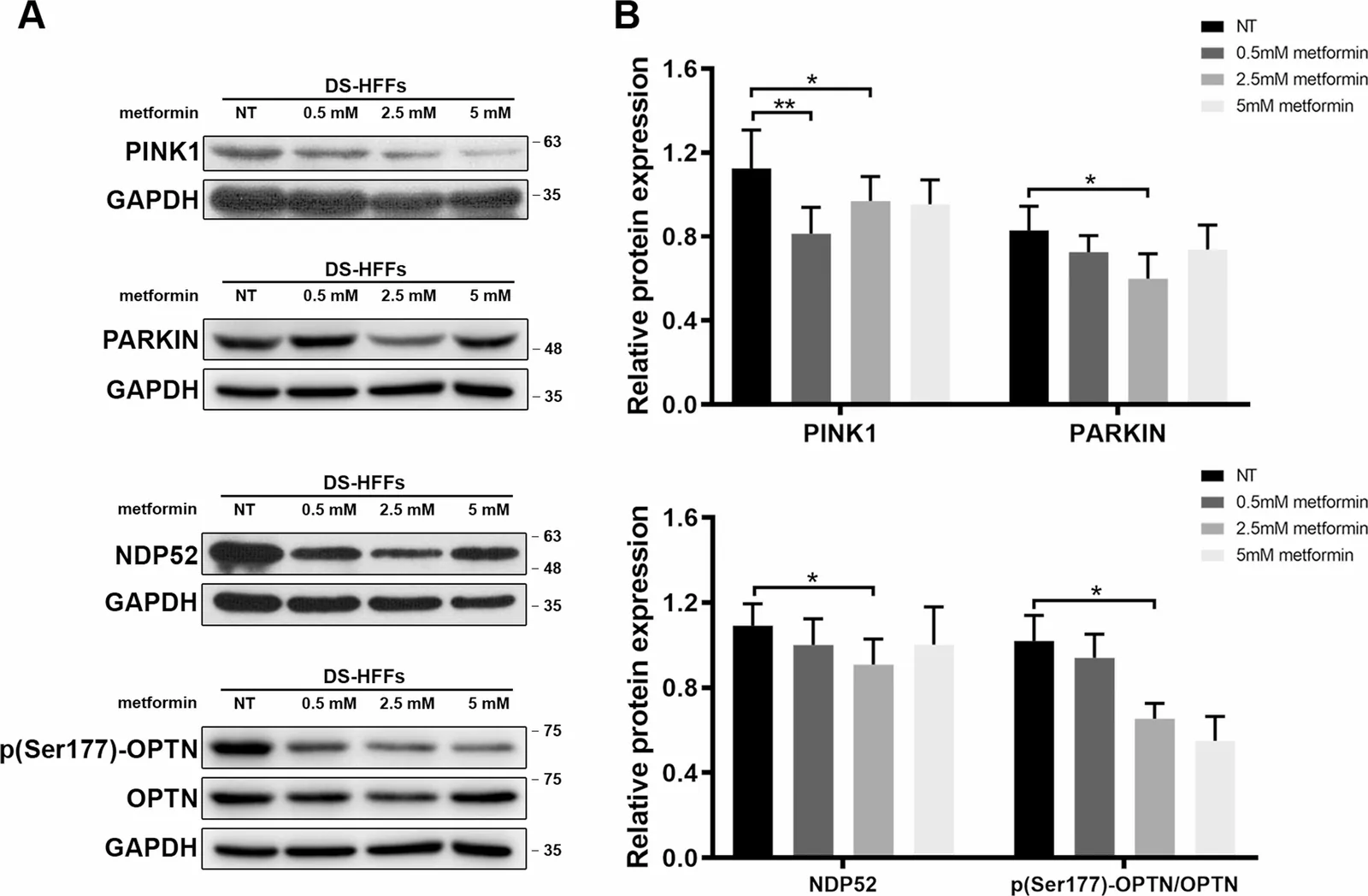
Metformin reduces PINK1, PARKIN, NDP52, and p(Ser177)-OPTN/OPTN ratio in trisomic fibroblasts.** (A)** Representative immunoblotting showing PINK1, PARKIN, NDP52, p(Ser177)-OPTN and total OPTN in DS-HFFs following metformin treatment. **(B)** Densitometric quantification from at least four independent experiments for each protein. Data are expressed as mean ± SEM of five DS-HFFs cultures. GAPDH was used as a loading control. DS-HFFs: trisomic fetal fibroblasts. *p ≤ 0.05; **p ≤ 0.01, ***p ≤ 0.001

We therefore assessed the effects of metformin treatment on key mediators of mitophagy to determine whether the reduced accumulation of damaged mitochondria previously reported in DS-HFFs [29] is associated with the modulation of mitophagy machinery. Treatment with 2.5 mM metformin resulted in reduced levels of the mitophagy-related proteins PINK1, PARKIN, and NDP52 **(**Fig. 7**)**. We next assessed the levels of OPTN phosphorylation at serine 177 (Ser177) that promotes binding to polyubiquitinated mitochondria [40]. The ratio of phosphorylated OPTN to total OPTN was reduced following metformin treatment (Fig. 7).

**Fig. 8:**
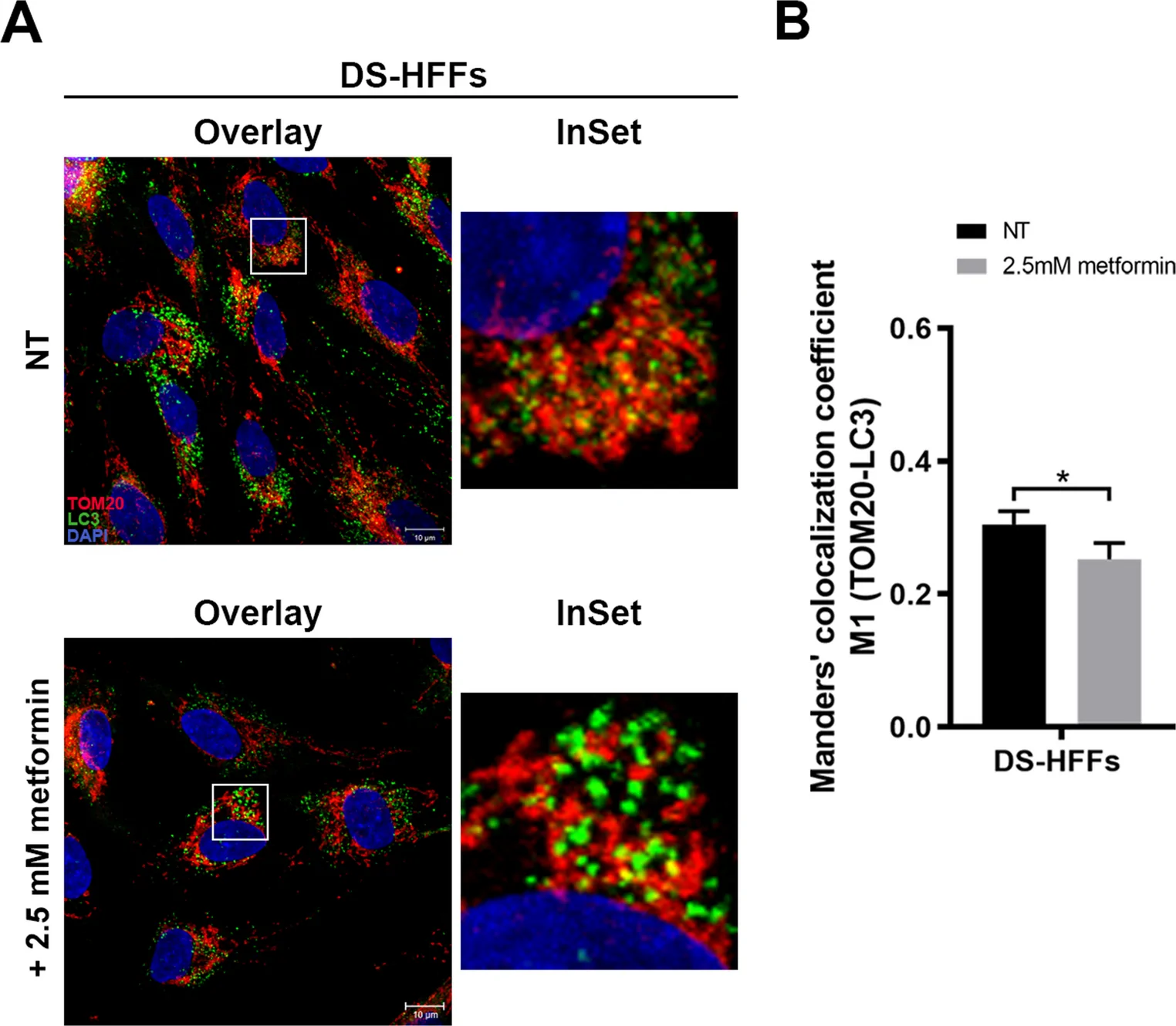
Metformin reduces colocalization of mitochondria with LC3-positive autophagosomes in trisomic fibroblasts.** (A)** Representative confocal images of DS-HFFs under basal conditions and after metformin treatment, stained for the mitochondrial marker TOM20 (red) and LC3 (green), and their relative InSet image. Nuclei are labelled with DAPI (blue). Scale bar: 10 μm. **(B)** The graphs represent the Manders’ colocalization coefficient M1 of TOMM20-LC3 overlap, calculated using the JACoP plugin of ImageJ. The analysis was performed on two independent DS-HFFs cultures treated with metformin. Fifty randomly selected cells per sample were analyzed. DS-HFFs: trisomic fetal fibroblasts. *p ≤ 0.05

To further assess changes in mitochondria-autophagosome association, we quantified TOM20–LC3 colocalization. Metformin treatment (2.5 mM) significantly reduced TOM20–LC3 colocalization (Fig. 8), indicating improved autophagosome processing and a shift away from mitochondrial sequestration.

**Fig. 9:**
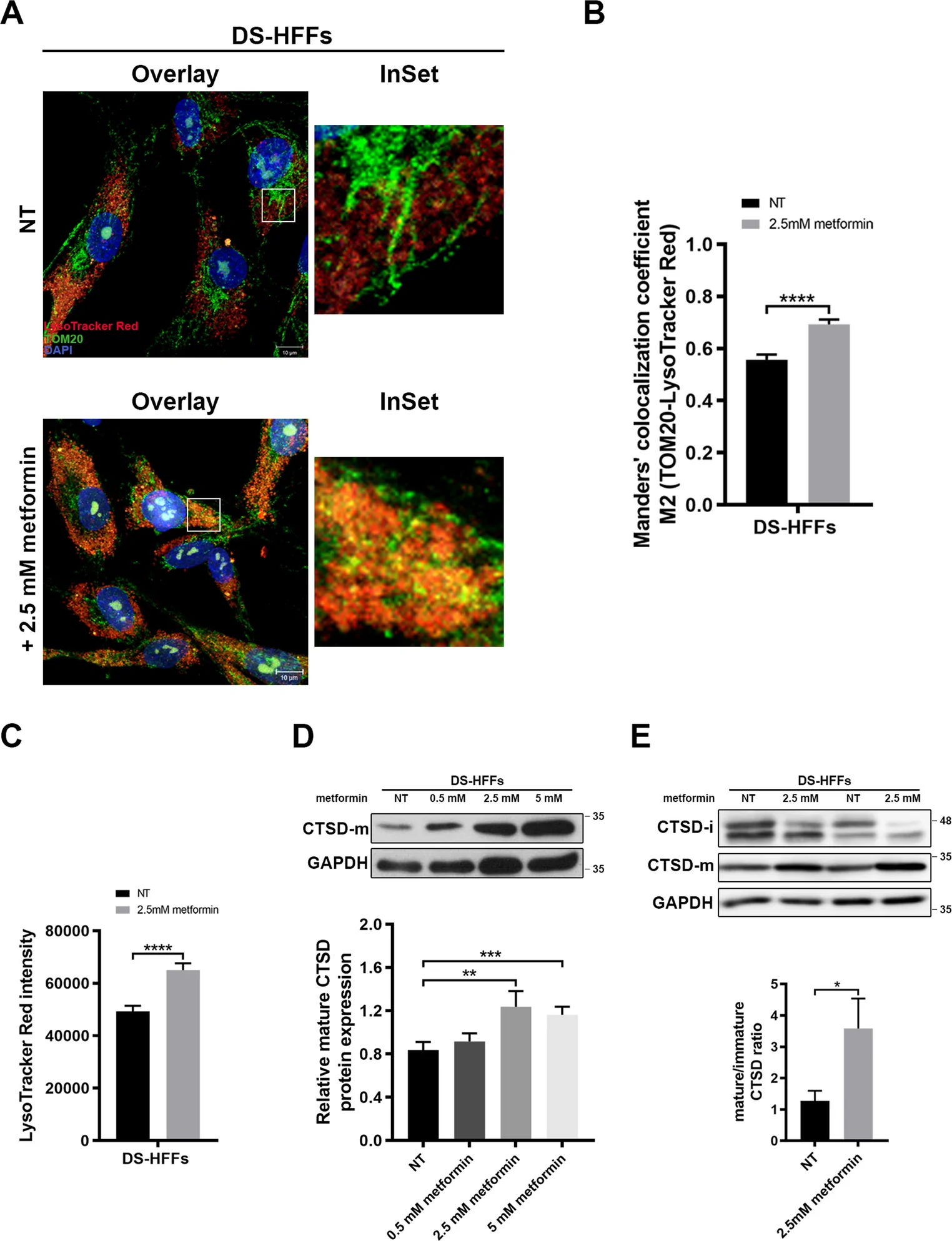
Metformin enhances lysosomal function in trisomic fibroblasts.** (A)** Representative confocal images of DS-HFFs under basal conditions and after metformin treatment, stained with the mitochondrial marker TOM20 (green) and LysoTracker Red, and their relative Inset images. Nuclei are labelled with DAPI (blue). Scale bar: 10 μm. (**B**) The graphs represent the Manders’ colocalization coefficient M2 of TOMM20-LysoTracker Red overlap, calculated using the JACoP plugin of ImageJ. **(C)** Quantification of LysoTracker Red fluorescence intensity in untreated and metformin-treated DS-HFFs. Data represent relative mean values ± SD from at least two independent experiments. Analyses were performed on two independent trisomic cultures, with fifty randomly selected cells per sample. (**D**) Representative immunoblots and densitometric quantification of the mature form of CATHEPSIN D following metformin treatment. (**E**) Representative immunoblots and densitometric quantification of the mature/immature CATHEPSIN D ratio following metformin treatment. For CATHEPSIN D, “-i” indicates the immature form, and “-m” indicates the mature form. Data are expressed as mean ± SEM of five DS-HFF cultures. GAPDH was used as a loading control. DS-HFFs: trisomic fetal fibroblasts. **p ≤ 0.01, ***p ≤ 0.001 ****p ≤ 0.0001

To assess whether metformin impacts the lysosomal arm of the degradative process in trisomic fibroblasts, we analyzed lysosome-mitochondria association using LysoTracker Red and TOM20 staining following treatment with 2.5 mM metformin. Metformin treatment increased TOM20 and LysoTracker Red colocalization in DS-HFFs **(**Fig. 9A, B**)**, consistent with enhanced association of mitochondrial cargo to acidic degradative compartments. Importantly, we observed an increase in LysoTracker Red fluorescence intensity following metformin treatment (Fig. 9C), consistent with altered lysosomal abundance and/or acidification. At the biochemical level, metformin increased the mature, processed form of cathepsin D (Fig. 9D), a lysosomal protease required for intra-lysosomal degradation. This protein was unaffected by trisomy 21 [41] but metformin increased its processing, as demonstrated by the increased ratio between the mature and immature forms at 2.5mM concentration (Fig. 9E).

**Fig. 10:**
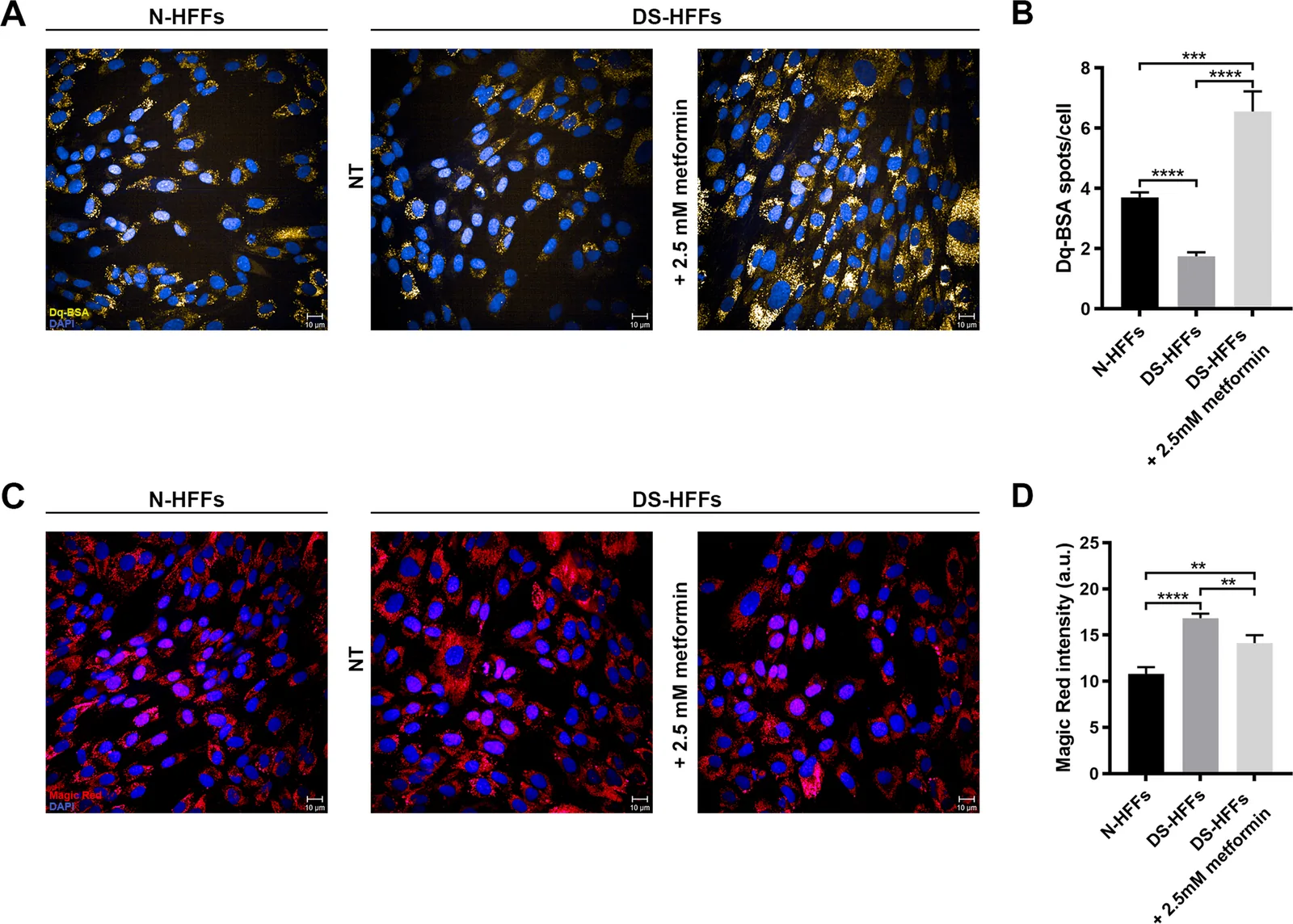
Metformin rescues lysosomal proteolytic activity of trisomic fibroblasts. (**A**) Lysosomal degradation activity was quantified using Dq- Red-BSA in N-HFFs and in DS-HFFs, with and without metformin. Nuclei are labelled with DAPI (blue). Scale bar: 10 μm. (**B**) The graph illustrates quantification of Dq-BSA spots-per-cell. (**C**) Cellular Cathepsin B activity was visualized in N-HFFs and in DS-HFFs, with and without metformin, by using the Magic Red reagent. Nuclei are labelled with DAPI (blue). Scale bar: 10 μm. (**D**) The graph represents the intensity of the Magic Red (arbitrary units). Data represent relative mean values ± SEM from at least two independent experiments. Analyses were performed on two independent trisomic cultures and two independent euploid cultures. At least fifty randomly selected cells per sample were analyzed. N-HFFs: euploid fetal fibroblasts; DS-HFFs: trisomic fetal fibroblasts. ** *p* ≤ 0.01, *** *p* ≤ 0.001, **** *p* ≤ 0.0001

Since LysoTracker staining is influenced by lysosomal volume and pH, both of which may be increased in dysfunctional lysosomes, we directly assessed lysosomal proteolytic activity by evaluating the release of bright fluorescent fragments from Dq-BSA, a probe degraded by proteolysis in lysosomes [42]. High content screening (HCS) analysis was performed in N-HFFs, DS-HFFs and DS-HFFs treated with metformin and quantification of fluorescent spots revealed a significative reduced lysosomal proteolytic capacity in trisomic cells that was rescued after metformin treatment (Fig. 10A, B). This observation supports enhanced lysosomal proteolytic capacity following metformin treatment.

To determine whether cathepsin activities were inhibited by protease inhibitors in trisomic cells, we assessed cathepsin B activity using the Magic Red assay in HCS. Compared with N-HFFs, DS-HFFs exhibited higher activity of this exopeptidase, which was partially normalized after metformin treatment (Fig. 10C, D).

**Fig. 11:**
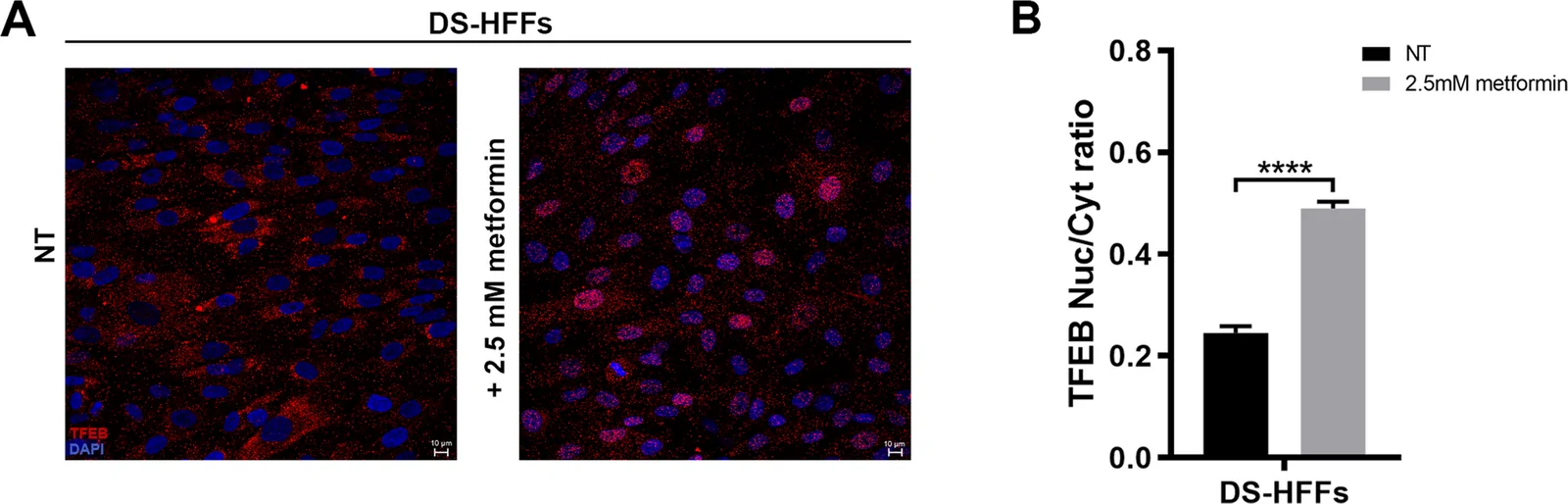
Metformin increases TFEB nuclear translocation in trisomic fibroblasts. (**A**) Representative confocal images of DS-HFFs, under basal conditions and after metformin treatment, stained for TFEB (red). Nuclei are stained with DAPI (blue). Scale bar: 10 μm. (**B**) Quantification of TFEB nuclear translocation expressed as the mean ratio ± SEM of nuclear to cytosolic fluorescence intensity (Nuc/Cyt ratio), measured using ImageJ from at least two experiments on two independent trisomic cultures. At least one hundred randomly selected cells per sample were analyzed. DS-HFFs: trisomic fetal fibroblasts; ****p ≤ 0.0001

The metformin-dependent activation of autophagy could be mediated by the Transcription factor EB (TFEB) activity, which mainly depends on its phosphorylation status and cytoplasm/nucleus shuttling [43]. Indeed, nuclear translocation is a hallmark of TFEB activation. By immunofluorescence staining, we found that metformin treatment increased the nuclear accumulation of TFEB (Fig. 11).

Collectively, these findings indicate that metformin partially restores the functional integrity of the autophagy–mitophagy–lysosome axis in trisomic fibroblasts, alleviating late-stage bottlenecks.

## Discussion

In this study, we identify a functional impairment of the mitophagic machinery in trisomic human fetal fibroblasts, characterized by sustained engagement of mitophagy-related proteins but inefficient mitochondrial clearance. While key components of the PINK1–PARKIN pathway are elevated at steady state, autophagic flux is reduced and delivery of mitochondria to acidic degradative compartments is compromised, indicating that mitophagic activation does not translate into effective organelle turnover.

The increased abundance of PINK1, PARKIN, OPTN, and NDP52 levels likely reflects a compensatory response to mitochondrial dysfunction, in agreement with our previous demonstration of a significant decrease of mitochondrial membrane potential and bioenergetic impairment in trisomic cells [29]. Importantly, mitochondrial depolarization represents a prerequisite for PINK1 stabilization on the outer mitochondrial membrane and subsequent PARKIN recruitment, placing this alteration upstream of canonical PINK1-PARKIN-mediated mitophagy [16].

These findings parallel reports of heightened mitophagy signaling in the Ts2Cje mouse brain, especially in the early postnatal stages (P0 and 1 month) [44], but contrast with previous studies reporting reduced mitophagy in other DS cell types [19, 45]. Together, these observations suggest that mitophagy dysregulation in DS is cell-type specific and reflects a complex imbalance rather than uniform suppression. Importantly, our data indicate that the defect does not reside at the level of mitophagy initiation but rather at later stages of autophagic processing.

Since mitophagy depends on proper functioning of the autophagic machinery [25], we examined proteins regulating autophagosome biogenesis and turnover. We focused on LC3 and the autophagy receptor p62, widely used markers of autophagic flux. LC3 is processed into LC3-I and then converted to LC3-II, which associates with autophagosomal membranes and serves as a marker of autophagy [46, 47]. Through its N-terminal binding domain, LC3 interacts with LIR-containing proteins [48], such as p62 [49], enabling the recruitment of autophagosomes to damaged mitochondria during mitophagy [17]. We found concomitant increases in LC3-II and p62 in DS-HFFs, together with reduced responsiveness to CCCP and attenuated flux in the presence of bafilomycin A1, providing a definitive biochemical validation of the stalled autophagic flux. Although elevated LC3-II alone might reflect either increased autophagosome production or a downstream block, the simultaneous rise in p62 clearly points to a defect in late-stage autophagic processing [37]. This interpretation aligns with previous evidence of impaired autophagosome–lysosome fusion in trisomic cells [50].

Mitophagy culminates in the recruitment of damaged mitochondria to lysosomes for degradation, making lysosomal function and positioning a critical determinant of mitochondrial quality control. Previous studies have reported abnormalities in lysosomal morphology, positioning, and acidification in trisomic cells [41, 51, 52], raising the possibility that defects at the level of the lysosomal compartment contribute to inefficient mitochondrial clearance. In line with this hypothesis, trisomic fibroblasts exhibited markedly increased LysoTracker Red fluorescence, consistent with altered lysosomal acidification and/or redistribution [41]. Such changes may contribute to the inefficient clearance of mitochondria observed in trisomic fibroblasts.

We observed elevated mitochondria–autophagosome colocalization combined with reduced basal mitochondria–lysosome association, further indicating that damaged mitochondria are sequestered but inefficiently delivered to degradative compartments. To overcome potential biases related to increased mitochondrial mass or lysosomal abundance in trisomic cells, we employed thresholded Manders’ coefficients instead of standard correlation metrics. This approach allowed us to decouple the quantification of organelle sequestration from signal density, providing a more accurate measure of the fraction of mitochondria effectively engaged by the autophagic and lysosomal compartments.

Together, these findings indicate that, although mitochondria in trisomic fibroblasts can be recognized and recruited toward acidic compartments, downstream processing is inefficient. Although the addition of CCCP is a standard method for inducing mitophagy in cells, it has been shown to disrupt the LysoTracker signal and cell proliferation when compared to the overexpression of UCP1, which induced a comparable level of mitochondrial depolarization without affecting LysoTracker signal or cellular proliferation [53]. Indeed, CCCP-induced lysosomal perturbation may contribute to the lack of a clear increase in TOM20–LysoTracker colocalization in euploid fibroblasts. Therefore, results obtained after induction of mitophagy with mitochondrial uncouplers should be interpreted with caution, because of their potential impact on lysosomes. However, when integrated with the accumulation of autophagosomes, reduced autophagic flux, and elevated mitophagy-related proteins described above, the data support the presence of a bottleneck at late stages of the autophagy–mitophagy–lysosome pathway. This impairment likely contributes to the accumulation of dysfunctional mitochondria and autophagy-related components (LC3-II, p62, PINK1, PARKIN, OPTN, and NDP52), in agreement with previous observations in cells and tissues from individuals with Down syndrome [19, 50].

Given our previous observations that metformin improves mitochondrial morphology and function in trisomic fibroblasts [29], we next examined whether this compound could also modulate autophagy-related processes in these cells. Metformin has been reported to exert context-dependent effects on autophagy, most commonly promoting autophagic flux through AMPK-dependent pathways and modulation of mTOR signaling. Numerous signaling pathways converge on this response, including AMPK-related cascades and downstream regulators such as mTOR, a central inhibitor of autophagy [54–58]. Notably, heightened mTOR activity has been consistently reported in DS cellular and animal models [19], suggesting that dysregulated mTOR signaling contributes to impaired autophagy in trisomic cells.

Treatment of trisomic fibroblasts with increasing concentrations of metformin for 72 h reduced mTOR phosphorylation at Ser2448, consistent with modulation of mTOR-associated signaling, a pathway known to inhibit autophagy. Importantly, the concomitant decrease of LC3-II and p62 in treated cells suggests improved autophagosome turnover rather than suppression of autophagosome formation. This interpretation aligns with previous studies reporting that metformin can modulate autophagy via AMPK-dependent pathways and mTOR signaling [59, 60]. For example, Gao et al. [61] demonstrated that metformin activates AMPK, suppresses mTOR phosphorylation, and decreases p62 accumulation in tumor cells. We acknowledge that alternative metformin mechanisms could explain the effects on autophagy [9, 62]. Additional studies have shown that metformin promotes autophagosome–lysosome fusion after spinal cord injury [63] and enhances autophagic processing in microglial cells [64].

Notably, while LC3-II accumulation has been observed at high metformin concentrations in certain tumor models [65], the decrease we observed in DS-HFFs suggests that the trisomic cellular environment—characterized by elevated yet inefficient basal autophagic activity—responds to metformin by improving downstream degradative steps rather than by further inducing autophagosome formation. These observations highlight the importance of cellular context in shaping metformin’s effects on the autophagy–mitophagy axis and support a model in which the drug alleviates pre-existing bottlenecks in autophagic turnover.

Together, these data indicate that metformin is associated with improved autophagic processing in trisomic fibroblasts, resulting in decreased accumulation of damaged mitochondria and a reduced requirement for sustained engagement of mitophagy-related components. This mechanistic framework is consistent with the previously reported effects of metformin on mitochondrial quality control in Down syndrome [29]. Furthermore, the observed decrease in OPTN phosphorylation at Ser177 and in mitophagy-related protein levels is consistent with alleviation of activation pressure on a previously overloaded system, paralleling the improved autophagic turnover observed in the preceding experiments.

Because completion of mitophagy depends on lysosomal competence, we examined whether metformin influences lysosome-related properties and mitochondria-lysosome interactions in trisomic fibroblasts. Crucially, metformin enhances mitochondria–lysosome association, increases LysoTracker fluorescence intensity, and promotes maturation of cathepsin D. The significant increase in the mature form of Cathepsin D and of its processing rate following metformin treatment serves as a key indicator of rescued proteolytic competence.

The improvement of lysosomal function in metformin-treated trisomic fibroblasts was further demonstrated by the increase in Dq-BSA fluorescence, indicating a rescue of the general degradative capacity of lysosomes rather than a specific lysosomal enzymatic activity. Indeed, we previously demonstrated that the levels of mature Cathepsin D were not different between euploid and trisomic samples [41] whereas other authors reported decreased levels in trisomy 21 fibroblasts [51]. Instead, CTSB, a lysosomal exopeptidase, was more active in DS cells, as demonstrated by the Magic Red assay, both in this work and in other models [51]. This could indicate a compensatory effect aimed at counteracting the inhibition of the autophagy pathway and the overload of upstream mitophagy mediators. Overall, metformin can restore the balance between lysosomal proteolytic activities.

This evidence indicates that metformin does not merely facilitate the routing of mitochondria to acidic compartments but actively restores the degradative environment necessary for organelle clearance. Lysosome-mediated AMPK activation has been described [66], although in the present study we have not observed activation of the AMPK substrates, possibly because AMPK is already activated in untreated DS-HFFs, likely reflecting a chronic energetic stress response. Other mechanisms could also be involved, as metformin has been shown to recognize different lysosomal molecular targets [9]. For example, low-dose metformin has been reported to engage lysosomal AMPK signaling via PEN2 [67]. In addition, lysosome-dependent signaling pathways have been implicated in metformin-dependent longevity responses in *C. elegans* [68], reinforcing the notion that lysosomes may act as critical effectors of metformin action. Additional evidence supports an effect of metformin on late stages of autophagy. Tai et al. [69] reported that metformin accelerates autophagic flux in vascular smooth muscle cells by increasing autophagosome-lysosome fusion, upregulating LAMP1, and promoting lysosomal functionality.

The recovery of lysosomal function could also be ascribed to TFEB activation, as demonstrated by the increased nuclear shuttling of this transcription factor in trisomic cells treated with metformin. TFEB acts as a key regulator of genes involved in lysosomal biogenesis and autophagy [70]. Its activity is tightly controlled by its phosphorylation status. In its phosphorylated form, TFEB remains inactive and is predominantly localized in the cytoplasm. In response to cellular stress, TFEB undergoes dephosphorylation through kinase inhibition and phosphatase activation, leading to its activation and subsequent translocation to the nucleus. Metformin has been shown to exert protective effects against hepatic steatosis and insulin resistance by significantly restoring TFEB activity [71].

Taken together, these findings support a two-tier model to explain mitochondrial dysfunction in trisomy 21. The first tier consists of a mitochondrial program altered by gene-dosage—potentially involving key regulators such as NRIP1—which may act as an initiating event sustaining chronic mitochondrial stress. Superimposed on this, there is a second tier, characterized by a limited resolution capacity of the autophagy–lysosomal system, which creates a late-stage bottleneck and impairs effective mitophagic clearance. Pharmacological enhancement of degradative capacity, as observed with metformin, partially restores mitochondrial turnover and reduces the accumulation of mitophagy-related intermediates.

While we acknowledge that direct pH-sensitive mitophagy reporters provide real-time turnover measurements, the robustness of our conclusions is underpinned by an integrated characterization based on a triad of independent evidence: quantitative Manders’ imaging, biochemical flux validation with bafilomycin A1, and the assessment of terminal lysosomal proteolytic activity. The convergence of these complementary readouts renders our interpretation of the functional saturation of the degradative machinery, and its pharmacological rescue by metformin, extremely robust and less susceptible to the technical confounds of single-probe assays.

Overall, in trisomic fibroblasts, increased mitophagy-related markers do not indicate efficient mitophagy, but rather accumulation of unresolved intermediates due to impaired lysosomal clearance. Metformin does not simply stimulate upstream mitophagy; it appears to relieve a late-stage lysosomal bottleneck, restoring degradative competence and thereby reducing the accumulation of mitophagy-related components.

## Data Availability

The manuscript has non associated data.
